# Same-Day Discharge After a Minimally Invasive Colectomy: A Successful Approach to Patient Selection

**DOI:** 10.7759/cureus.67250

**Published:** 2024-08-19

**Authors:** Daniel Aillaud-De-Uriarte, Luis A Hernandez-Flores, Andrea Hernandez-Moreno, Philip N Zachariah, Ria Bhatia, Jorge Rodriguez-Gaytan, Diego Marines-Copado

**Affiliations:** 1 Division of Colon and Rectal Surgery, Houston Methodist Willowbrook Hospital, Houston, USA; 2 Center for Bioethics, Harvard Medical School, Boston, USA; 3 Department of Gastroenterology, Drexel University College of Medicine, Philadelphia, USA; 4 Department of Epidemiology and Biostatistics, The University of Texas at Austin, Austin, USA

**Keywords:** same-day discharge, readmission, colorectal surgery, pain management, colectomy

## Abstract

Background: Enhanced Recovery After Surgery (ERAS) protocols have been shown to decrease inpatient length of stay (LOS) and improve surgical outcomes in elective abdominal colorectal procedures. Discharging a patient home after a minimally invasive colectomy on the same calendar day is a multifactorial decision that takes into account the patient’s decision and baseline condition, social factors, intraoperative findings, and postoperative recovery status. The aim of this study is to evaluate the outcomes of same-day discharge (SDD) following minimally invasive colectomy within an ERAS protocol in a community hospital setting in Houston, Texas.

Methods: In this retrospective cohort study, all consecutive elective cases were performed by a single surgeon from April 2022 to April 2023. This retrospective analysis aims to report a single senior surgeon's experience of the safety, feasibility, and benefits of same-day discharge after minimally invasive colectomy in preselected patients. Same-day discharge was defined as a discharge on the same calendar day without an overnight stay. Differences between specific groups were compared using the Fisher's exact test and Mann-Whitney U test.

Results: Of 86 non-emergent colectomies, 41 patients (47.7%) were successfully discharged on the same day. The median age of the patients was 63.50 years (interquartile range (IQR) 18). The cohort included 37 females (43%) and 49 males (57%). The median LOS was one day. The median operating time was 148.50 minutes (IQR 68.25). The median intraoperative fluid usage was 1500 mL (IQR 36.25), and the median estimated blood loss (EBL) was 25 mL (IQR 36.25). No readmissions among the SDD patients (0%), while three readmissions were reported in patients who stayed overnight (3.4%).

Conclusion: Same-day discharge after a minimally invasive colectomy is feasible when there is a well-established ERAS protocol and there is adequate education for patients and staff. Adequate patient selection is crucial. Patients with multiple comorbidities and a lack of a support network are not suitable candidates.

## Introduction

Enhanced Recovery After Surgery (ERAS) has been demonstrated to decrease inpatient length of stay (LOS) and improve surgical outcomes after elective minimally invasive major abdominal colorectal procedures, including partial or subtotal colectomy, ileostomy, and colostomy reversal [1-3].

Same-day discharge (SDD) has multiple benefits across different categories. In 2019, a survey assessing SDD patients’ satisfaction found that patients with LOS of 0 days reported higher satisfaction compared to those with any other LOS. Most patients feel they are active decision-makers in their care and discharge [4]. Past literature shows improvement in patients' quality of life with the ERAS protocol, as well as a sooner return to their daily activities. Additionally, patients are less exposed to potential nosocomial infections and narcotics [5, 6]. This strategy improves resource utilization and is cost-effective [7, 8]. Our healthcare system benefits greatly from SDD as hospital beds become available to other patients who require inpatient care [9]. The challenge of SDD lies in the appropriate selection of the patients. The primary concern in instituting this protocol is the potential increase in complications and/or readmissions. Patients' education is fundamental. 

This study aims to report our experience of the SDD protocol in colorectal surgery.

## Materials and methods

A 12-month retrospective cohort study was performed, which included 86 patients admitted to the Houston Methodist Willowbrook Hospital, a community hospital in Houston, Texas, between April 2022 and April 2023. The chosen study period allowed for a comprehensive collection of cases, ensuring sufficient data for description. This community hospital setting was selected due to its diverse patient population, providing a representative sample of the local demographics and healthcare patterns.

The study investigated the selection, safety, feasibility, and outcomes of discharging patients who underwent minimally invasive colectomy on the same day of the surgery. Equally, we aim to find a correlation between patient characteristics and surgical markers in favor of or against a successful SDD. Equally, we aim to find a correlation between patient characteristics and surgical markers in favor of or against a successful SDD.

The initial patient population included 102 patients diagnosed with colon and rectal neoplasia, diverticulitis, and inflammatory bowel disease who underwent an elective colectomy. After implementing our exclusion criteria, 86 patients remained. Inclusion criteria included patients who underwent the following elective procedures: low anterior resection (LAR), sigmoidectomy, right and left hemicolectomy, segmental colectomy, diagnosis of colorectal masses/cancer, inflammatory bowel disease, and diverticulitis. Exclusion criteria included emergency procedures, rectopexy, transanal minimally invasive surgery (TAMIS), loop ileostomy, and colostomy reversal. Same-day discharge was defined as a discharge on the same calendar day with no overnight stay [10]. These patients were monitored via calls or messages until their follow-up appointment. All cases were performed by a single colorectal surgeon.

Differences between specific groups were compared using Fisher's exact test for categorical variables (sex, type of surgery, comorbidities, diagnosis, readmission, etc.). The Mann-Whitney U test was used for our continuous variables (operating time, intravenous fluids, estimated blood loss (EBL)), as our population was not normally distributed. A p-value of <0.05 was considered statistically significant. Data were collected on a private server (REDcap electronic data capture tools powered by Vanderbilt, Nashville, TN, and supported in part by the National Institutes of Health, Bethesda, MD) through a retrospective chart review from the electronic medical records (EMR) located in the hospital’s database. Statistical analysis was performed using IBM SPSS Statistics software for Windows version 27 (IBM Corporation, Armonk, NY). 

Determining a patient's suitability to be considered for SDD after minimally invasive colorectal surgery involves comprehensive, multifactorial decision-making that takes into account the patient’s condition before the surgery, during the procedure, and in the recovery room, as well as the patient's desire. (Table 1). The surgeon counseled the patients and their families about the procedure and discharge process during the preoperative visit. Questions and expectations were addressed at this time. Adequate home support, including social support, access to telecommunication, and distance from their home to the hospital, was assessed as well. A transversus abdominis plane (TAP) block was performed by the anesthesiologist preoperatively [11]. The patients also received oral gabapentin, acetaminophen, and celecoxib. Bowel preparation was done using low-volume split dose prep with the addition of oral antibiotics (metronidazole 500 mg per oral (PO) thrice a day (TID) and neomycin 1 g PO single dose). When discharged, patients received ambulatory pain control, including hydrocodone and acetaminophen.

**Table 1:** Patient selection criteria for same-day discharge (SDD) NSAID: non-steroidal anti-inflammatory drugs; ICG: indocyanine green; DM2: type 2 diabetes; HTN: hypertension

Eligibility criteria for SDD	Details
Eligible candidates	- Adults
	- Independent
	- Adequate home support
	- <1 hour travel from hospital
	- Telephone or mobile device access
Ineligible candidates	- Allergies to bupivacaine and NSAIDs
	- Cognitive impairment
	- Severe comorbidities
	- Coagulopathy
	- Uncontrolled DM2 or HTN
	- Chronic opioid use
	- >10 mg prednisone
	- Diverting loop ileostomy plan
	- Patient preference
Intraoperative requirements	- Adequate ICG and normal leak tests
	- No excessive anastomosis tension
	- Proper washout of abscess pockets
Intraoperative complications	- Excessive bleeding - Prolonged adhesions lysis
Postoperative milestones	- Tolerance of PO liquids
	- Normal urinary function
	- Sustained ambulation
Postoperative complications	- Hemodynamic instability
	- Persistent nausea/vomiting
	- Uncontrolled pain

Finally, in the recovery room, patients were assessed for PO liquid tolerance, independent ambulation, and urination. They needed to be hemodynamically stable, have adequate pain, and have antiemetic control with minimal IV pain medication. Bowel movements or flatus were not needed prior to discharge. 

Assessment of the patients in the recovery room and their discharge was successfully established with the help of a hospitalist physician who participated actively in communication with the colorectal surgeon. Patients received a follow-up phone call the next morning, and then they were seen at the clinic two weeks after surgery. Patients communicated their questions through their medical records online platform or by phone. 

This retrospective study was approved by the Research Institute Committee, Houston Methodist Willowbrook Hospital, Houston, TX, with approval number PRO00037705, and conducted in accordance with the ethical standards of the Declaration of Helsinki.

## Results

After reviewing 102 surgeries within the 12-month study period, 86 non-emergent colectomies were performed, 41 patients were discharged on the same day (47.7%), and 45 stayed overnight (52.3%). In our population, the median age was 63.50 years (IQR 18), the median BMI was 27.59 kg/m^2^ (interquartile range (IQR) 7.5), the median operating time was 148.50 minutes (IQR 68.25), the median intraoperative fluid was 1500 mL (IQR 719), and the median EBL was 25 mL (IQR 36.25). The LOS showed a median of one.

The most frequently undertaken surgeries were as follows: LAR in 46 patients (50% SDD rate), right colectomy in 28 patients (50% SDD rate), left hemicolectomy in four patients (25% SDD rate), total colectomy in one patient (0% SDD rate), transverse colectomy in two patients (0% SDD rate), segmental colectomy in three patients (67% SDD rate), and Hartmann’s procedure in two patients (50% SDD rate) (Table 2). The most common surgery in our SDD population was LAR (26.7%). Eighty-three of the cases were performed robotically (44.2% SDD), one laparoscopically (1.2% SDD), and two by laparotomy (0% SDD). Indications for the procedures were classified as malignant (10.5% SDD vs. 19.8% non-SDD) and non-malignant (37.2% SDD vs. 32.6% non-SDD). 

**Table 2:** Description of surgeries Age, OP time, EBL, and IO fluids are averages n: number; SDD: same-day discharge; OP time: operating time; EBL: estimated blood loss; IO fluids: intraoperative fluids

Type of surgery	n	Robotic (n)	SDD (%)	Age (years)	Diagnosis		Male:female	OP time (min)	EBL (mL)	IO fluids (mL)
					Non-malignant	Malignant				
Low anterior resection and sigmoidectomy	46	45	26.7	59.4	31	15	1:1	162.9	48.0	1399.5
Right hemicolectomy	28	26	16.3	62.1	21	7	7:1	136.6	59.5	1504.4
Left hemicolectomy	4	4	25	48.0	3	1	1:3	176.0	66.3	1550.0
Total colectomy	1	0	0	48.0	1	0	1:0	214.0	50.0	1000.0
Transverse colectomy	2	2	0	54.5	2	1	1:1	214.5	127.5	2625.0
Segmental colectomy	3	3	2.3	63.0	1	2	1:2	141.7	20.0	1566.7
Hartmann's procedure	2	2	1.2	68.0	2	0	1:1	249.0	175.00	1900.0

The distribution of the American Society of Anesthesiologists (ASA) classification showed 0% ASA I, 52.3% ASA II, 44.2% ASA III, and 3.5% ASA IV patients. 

The prevalence of comorbidities and substance usage in the SDD patients vs. non-SDD patients were compared. The patients discharged on the same day showed type 2 diabetes in eight patients (9.3%), hypertension in 23 patients (26.7%), smoking in 20 (23.3%), and alcohol consumption in 20 (23.3%). More detailed information about these patients' demographics is shown in Table 3.

**Table 3:** Patients' characteristics SDD: same-day discharge; F: female; M: male; BMI: body mass index; DM2: type 2 diabetes mellitus; HTA: arterial hypertension; ASA: American Society of Anesthesiologist classification

	SDD (n = 41)	Non-SDD (n = 45)
Age (years)	57.3 (±13.2)	62.9 (±13)
Gender	F 24 (27.9%)	F 25(29.1%)
	M 17 (19.8%)	M 20 (23.3%)
BMI (kg/m^2^)	29.2 (±6.7)	27.2 (±5.8)
DM2	8 (9.3%)	13 (15.1%)
HTA	23 (26.7%)	25 (29.1%)
Alcohol	20 (23.3%)	25 (29.1%)
Smoking	20 (23.3%)	21 (24.4%)
Malignant diagnosis	9 (10%)	19 (20%)
Non-malignant diagnosis	34 (35%)	34 (35%)
ASA	1 → 0	1 → 0
	2 → 26	2 → 19
	3 → 15	3 → 23
	4 → 0	4 → 3

In the Mann-Whitney U Test, a comparison between the two groups (SDD and non-SDD) showed statistical significance in the operating time (p = 0.001) and EBL (p = < 0.001); nevertheless, intraoperative fluids failed to prove a true difference between groups (p = 0.093). In Fisher’s exact test, categorical variables of malignant diagnosis, type of surgery, sex, comorbidities (hypertension, type 2 diabetes), and substance consumption (tobacco, alcohol) failed to prove statistical significance (Table 4).

**Table 4:** Differences between SDD group and non-SDD group SDD: same-day discharge; OP time: operating time; EBL: estimated blood loss; IO fluids: intraoperative fluids; IQR: intraquartile range *Indicates that the p-value is <0.05, representing a statistically significant result **Indicates that the p-value is <0.001, representing a strong statistically significant result.

	SDD	Non-SDD	p-value
OP time	132.4 (±36.4)	174.8 (±179.8)	p = 0.001*
EBL	21 (IQR 15)	75.2 (IQR 50)	p = < 0.001**
OI fluids	1367 (±412.5)	1597.8 (±593.6)	p = 0.093

None of the readmissions within the 30 days following surgery happened on patients discharged within the same day. Three patients who stayed overnight (non-SDD) were readmitted, representing 3.4% of the group. The reasons for readmission included a small bowel obstruction on the seventh day post discharge, pyelonephritis, and hypotension likely due to an adrenal crisis in a patient who was on steroids.

## Discussion

Our investigation, spanning a 12-month period and encompassing 102 patients, with a final population of 86 patients who underwent non-emergent colectomies, provided significant insights into the new era of ambulatory colectomy. Among the cohort, 47.7% of patients were discharged on the same day, and 52.3% stayed overnight. The most common surgery performed in SDD patients was LAR (26.7%), with an average age of 59 years. None of the patients on the SDD protocol experienced complications or readmission within the 30 days following their surgery. The most common diagnosis among these patients was diverticulitis. Statistical significance was found regarding operating time and intraoperative fluids when comparing the SDD and non-SDD populations. A key step to the success of SDD was the assessment of patients in the recovery room prior to discharge, which was facilitated by the active communication between a hospitalist physician and the colorectal surgeon.

It is crucial to highlight that patient selection for the SDD protocol plays a pivotal role in its success. Our findings suggest that patients who were discharged on the same day tended to have less severe diagnoses and lower ASA scores. Although statistical significance was not achieved in these comparisons, this may be attributed to the relatively small sample size. Proper patient selection is essential for optimizing outcomes and ensuring the safety and effectiveness of the SDD protocol.

In an era characterized by the pursuit of healthcare optimization and efficient resource utilization, our findings support the SDD protocol. The increasing interest in ambulatory colectomy stems from its potential to improve pre- and postoperative care, change the conventional paradigms of overnight stays, and enhance patient outcomes [12]. As the literature has shown the feasibility and safety of the SDD protocol for select colectomy patients [13, 14], our results support these findings by providing data on surgical outcomes and patient demographics, underscoring the SDD protocol outcomes on the patient’s discharge.

Previously, SDD had been described in the colorectal field, from loop ileostomy closure to colectomy with anastomosis creation [15]. These studies suggested that adequate, unhindered communication between patients and the health care team is vital for SDD [16]. Usually, support and guidance during the early postoperative period are required by the patients who are discharged on the same day [17, 18].

A systematic review evaluated the success rate of SDD in four studies, with a notable success rate of 47% (range 22%-98%) [19]. Notably, the highest success rate was seen in small populations (41 patients, 98% success rate, and 2.5% complications), where the indication of surgery was diverticulitis in 85% and cancer in 15% of patients [14]. Larger studies demonstrated a very low success rate (2.5% of 36,526 patients), with a low readmission rate as well (2%) [4]. Notably, a study described inadequate pain control as the most common reason for SDD failure in their population (2.9% out of 8.3% failure) [20]. It has been established that SDD after colorectal procedures requires well-established communication between the surgeons, anesthesiologists, nurses, office personnel, office staff, the patient, and the family [21].

Opioid use and abuse is a challenging problem in the United States [22]. Pain control is an integral part of the ERAS protocol, and opioids are useful at times, but they also carry many undesired side effects. The benefit of using nonopioid analgesics has been established. Adequate analgesia facilitates early mobility, early return of bowel function, and decreased postoperative morbidity. Reducing or avoiding opioids in the perioperative period is one of the most important components of a successful SDD program [5].

This study has several limitations that should be considered when interpreting the results. It is a retrospective cohort analysis conducted at a single center with a relatively small sample size. All surgeries were performed by a single surgeon, which limits the generalizability of our findings and may introduce bias. Additionally, the lack of a control group prevents a direct comparison of outcomes between SDD and traditional postoperative care. These limitations may impact the robustness of our conclusions and underscore the need for further research. 

To our knowledge, there are very few centers currently performing major colorectal surgery in an ambulatory setting. This study contributes valuable data to a relatively unexplored area, potentially paving the way for broader adoption and further research into the safety and efficacy of SDD protocols in colorectal surgery.

## Conclusions

Coordinated effort among the medical team (surgeons, anesthesiologists, nurses, and hospitalist physicians) and a meticulous review of patients’ postoperative status is crucial to achieving successful results. This requires an extensive pre- and postoperative educational protocol and enhanced pathways for patients to follow up via electronic media (calls, messages). Based on the data presented herein, which demonstrate the safety and feasibility of the SDD colectomy protocol in our hospital setting, we truly believe that prospective and retrospective research with a wider population is now needed to compare this approach to conventional overnight postoperative care and to formally assess its potential benefits and limitations.
